# 293. Brain Abscess After Hematopoietic Cell Transplantation: A 15-Year Retrospective Review at Mayo Clinic

**DOI:** 10.1093/ofid/ofaf695.096

**Published:** 2026-01-11

**Authors:** Sergio L Alvarez Mulett, Sofia Molina Garcia, Estefania Gauto Mariotti, Nischal Ranganath, Aditya Shah

**Affiliations:** Mayo Clinic, Rochester, MN; Mayo Clinic, Rochester, MN; Mayo Clinic Rochester, Rochester, Minnesota; Mayo Clinic, Rochester, MN; Mayo Clinic, Rochester, MN

## Abstract

**Background:**

Hematopoietic cell transplantation (HCT) is associated with a net state of immunosuppression to enable graft acceptance and mitigate graft-versus-host disease risk^1^. Most of these patients require multiple prophylactic antimicrobials to prevent opportunistic infections during the post-transplant course^2^, including central nervous system (CNS) infections^3^. Brain abscesses are a form of CNS infection with a high incidence of morbidity and mortality^4^

**Figure:**
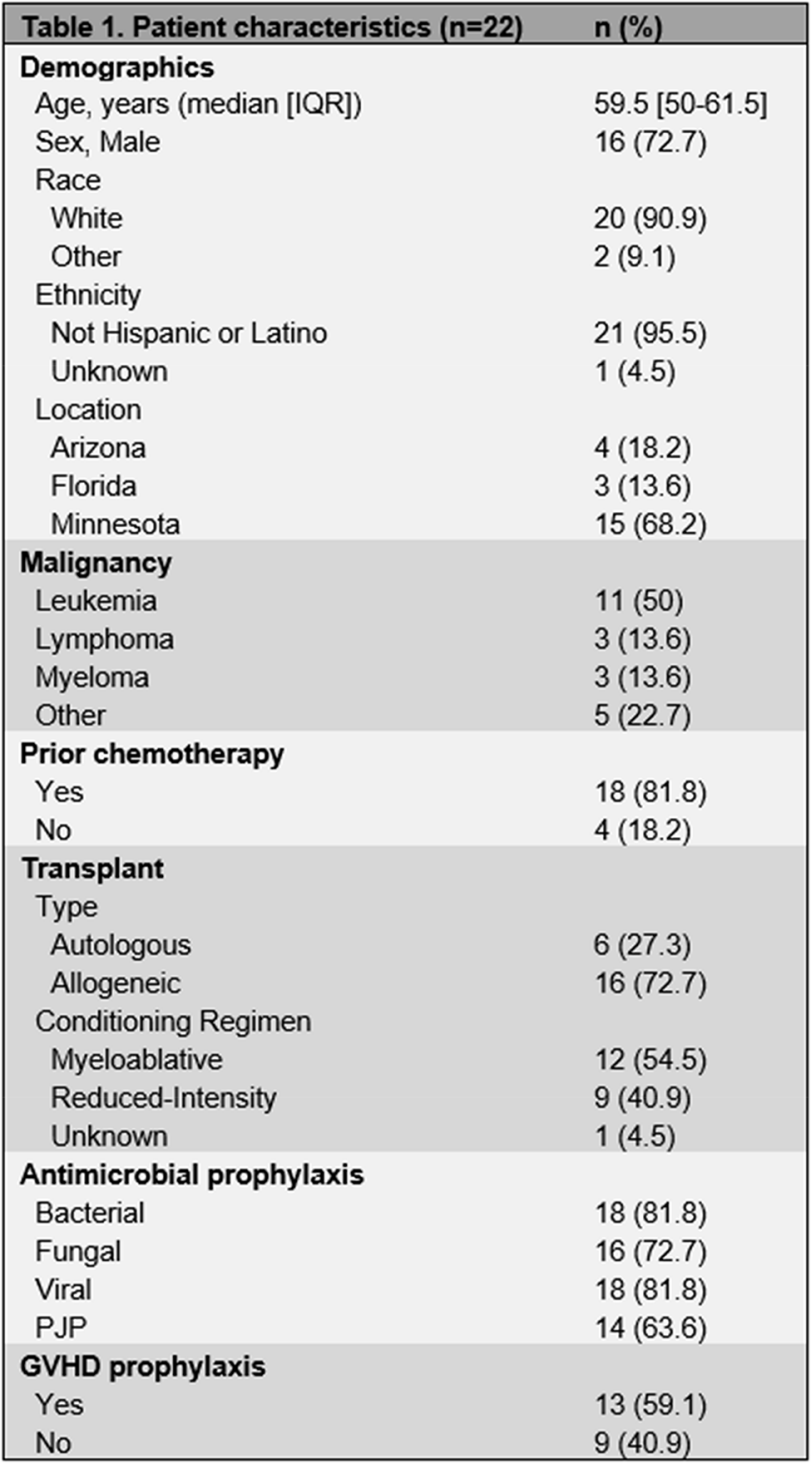

**Figure:**
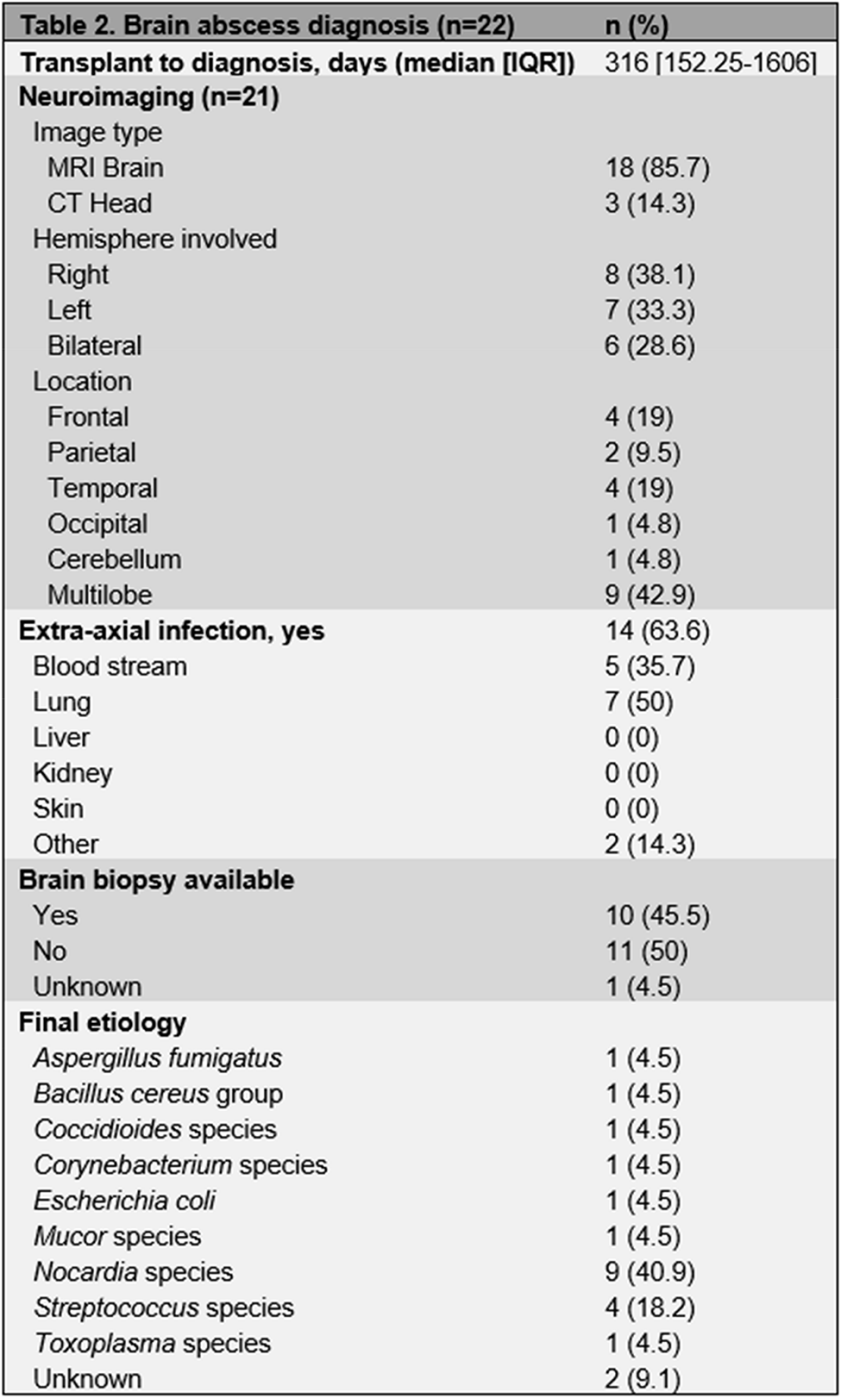

**Methods:**

We conducted a retrospective cohort study of adults diagnosed with brain abscess following HCT between January 2009 and December 2024, including patients from all Mayo Clinic sites (Arizona, Florida, and Minnesota). The study was approved by our institutional review board with a protocol number 24-011428. The data was extracted from the electronic medical records into a secure database in REDCap. Statistical analyses were conducted using R version 4.4.1 (R Foundation for Statistical Computing).

**Figure:**
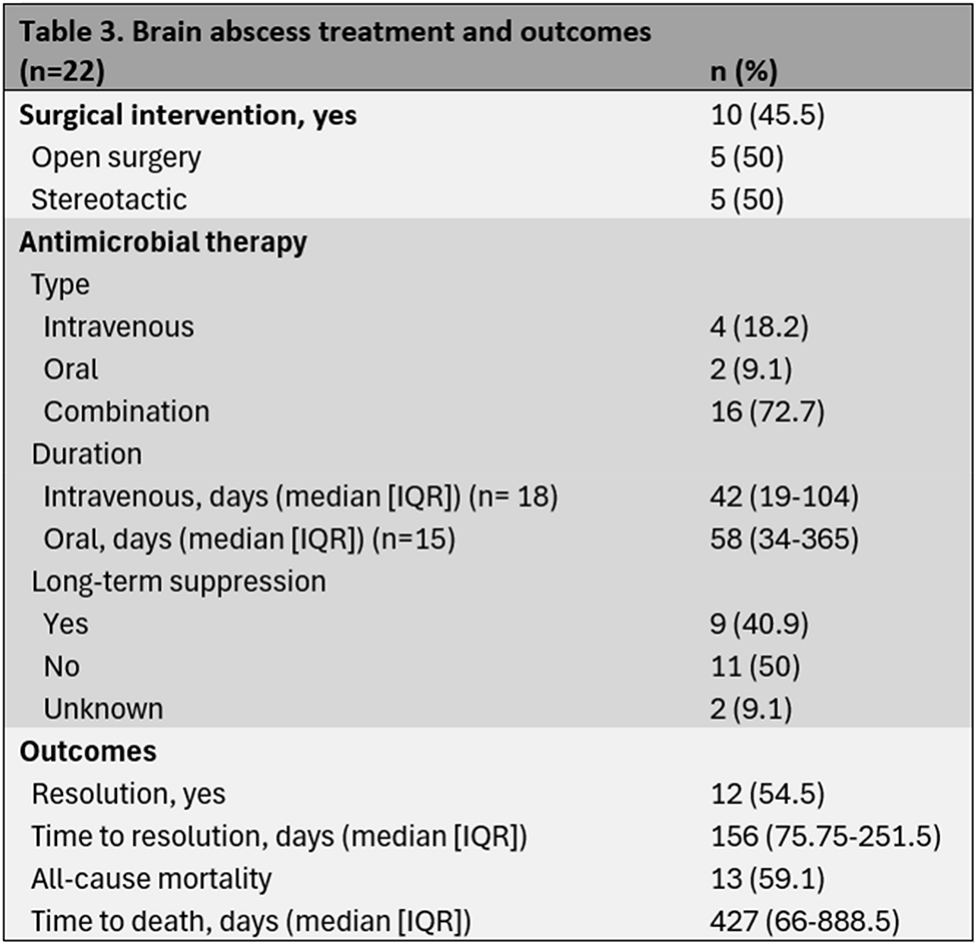

**Results:**

We identified 22 patients with brain abscess diagnosis following HCT. 50% of patients had leukemia. 81.8% of them received prior chemotherapy. 16 patients had prior allogeneic HCT, and 54.5% received a myeloablative conditioning regimen (table 1). 21 patients had neuroimaging available. More than one cerebral lobe was involved in 42.9% of the cases. 14 patients had extra-axial infection, the lung being the most frequently affected organ. *Nocardia* accounted for 40.9% of the brain abscesses (table 2). 45.5% underwent surgical intervention, and most of our patients were treated with a combination of intravenous and oral antimicrobials, many of them followed by long-term suppression. 54.5% of the brain abscesses resolved, with a median time to resolution of 156 days. All-cause mortality was 59.1%, with a median time to death of 540 days after brain abscess diagnosis (table 3).

**Conclusion:**

Although rare, brain abscess is a devastating complication of HCT, with high morbidity and mortality. The most common pathogen encountered was *Nocardia* species. The required treatment is usually extensive, and these patients often need long-term suppression. Further investigation is required to determine risk factors for specific pathogen infection, non-resolution, and mortality.

**Disclosures:**

All Authors: No reported disclosures

